# Investigating the cost‐effectiveness of three cessation interventions on a national scale using the Economics of Smoking in Pregnancy (ESIP) decision analytical model

**DOI:** 10.1111/add.15968

**Published:** 2022-06-14

**Authors:** Matthew Jones, Murray Smith, Sarah Lewis, Steve Parrott, Tim Coleman

**Affiliations:** ^1^ NIHR School for Primary Care Research University of Nottingham Nottingham UK; ^2^ Community and Health Research Unit University of Lincoln Lincolnshire UK; ^3^ Division of Epidemiology and Public Health University of Nottingham Nottingham UK; ^4^ Department of Health Sciences University of York York UK

**Keywords:** Cost‐effective, economic evaluation, pregnancy, smoking, smoking cessation, tobacco

## Abstract

**Aim:**

To measure the cost‐effectiveness of adding text message (TMB), exercise (EB) and abstinent‐contingent financial incentive‐based (CFIB) stop smoking interventions to standard smoking cessation support for pregnant women in England.

**Design:**

Modelling cost‐effectiveness outcomes by separately adding three cessation interventions to standard cessation care offered to pregnant women in England. English National Health Service Stop Smoking Services (NHS SSS) statistics from 2019 to 2020 were used for estimating the base quit rate. Intervention effectiveness and cost data for interventions were taken from trial reports. Cost‐effectiveness was derived using the economics of smoking in pregnancy (ESIP) model from a health service and personal social services perspective. Interventions were compared with each other as well as against standard cessation care.

**Setting:**

English NHS SSS.

**Participants/cases:**

A total of 13 799 pregnant women who accessed NHS SSS. Interventions and comparator; comparator: standard stop smoking support comprising behavioural intervention and an offer of nicotine replacement therapy (NRT). Three additive interventions were TMB, EB and CFIB.

**Measurements:**

Incremental cost‐effectiveness ratios per quality‐adjusted life‐years gained for both mothers and offspring over their life‐times; return on investment (ROI); and cost‐effectiveness acceptability curves (CEACs).

**Findings:**

The addition of any of the interventions compared with standard care alone was preferred, but only significant for the addition of CFIB, with the CEAC suggesting an at least 90% chance of being favoured to standard care alone. When compared against each other CFIB appeared to yield the largest returns, but this was not significant. The estimated ROI for CFIB was £2 [95% confidence interval (CI) = £1–3] in health‐care savings for every £1 spent by the NHS on the cessation intervention.

**Conclusions:**

For a health system which currently provides behavioural support and an offer of nicotine replacement therapy as standard stop smoking support for pregnant women, the greatest economic gains would be provided by operating an abstinent‐contingent financial incentives scheme alongside this.

## INTRODUCTION

Within‐pregnancy tobacco smoking remains a major global public health concern, with prevalence varying from 39% in Spain [1] to 12–14% in the United Kingdom, United States and Australia [2, 3, 4]. This exposes women and their offspring to both pregnancy‐related and long‐term risks from smoking [5, 6, 7, 8, 9]. Conservative estimates suggest that total annual health‐care expenditure for dealing with the impacts of smoking in pregnancy is USD110 million in the United States [10] and £23.5 million in the United Kingdom [11], with a UK child born to a smoking mother expected to generate £222 more in health‐care costs by age 5 years than a child born to a non‐smoking mother [12].

For any country, health‐care demand exceeds supply and hence rationing of resources is often required [13]. When outlining health‐care policy, economic evaluations (EEs) are now a vital component in the decision‐making process [14], including public health interventions [15]. This is especially prudent in England, where the National Institute of Health and Care Excellence (NICE) publishes a set of stringent requirements for EEs that is used to underpin their evidence‐based guidance [16]. They mandate that EEs should consistently take a life‐time time horizon, use generic health‐related quality of life outcomes [e.g. quality‐adjusted life‐years (QALYs)], take a UK National Health Service (NHS) and personal social services perspective, among a number of others. For EEs to be effective in aiding decision‐making this consistent approach is needed [17], yet most previous EEs of within‐pregnancy cessation interventions are incompatible with NICE guidelines [18], with time horizons limited to within‐pregnancy, pregnancy‐focused outcomes (e.g. complications avoided) and narrow perspectives. This impedes decision‐making; for example, drawing comparisons between the different evaluations to identify a preferred intervention from a set of competing alternatives is, at best, difficult.

When we planned this study, standard UK NHS support for smoking pregnant women was to offer behavioural support and nicotine replacement therapy [19]. More recently, three UK‐based randomized controlled trials (RCTs) have reported on interventions for encouraging pregnant women to stop smoking, which could become additional standard NHS cessation support; text message‐(TMB), exercise‐(EB) and abstinent‐contingent financial incentive‐based (CFIB) [20, 21, 22, 23, 24]. Each trial included EEs, but the inconsistent approaches did not meet NICE guidelines [16]. Therefore, we aimed to improve the evidence available on the cost‐effectiveness of TMB, EB and CFIB stop smoking interventions compared to standard smoking cessation support for pregnant women in England, such that the evidence meets UK decision‐making criteria.

## METHODS

### Objectives

Our primary aim was to evaluate the cost‐effectiveness of TMB, EB and CFIB cessation interventions compared to standard NHS care in England. Our objectives were to:

- programme the Economics of Smoking in Pregnancy (ESIP) decision analytical model with a cohort representing women who access NHS Stop Smoking Services (NHS SSS) while pregnant [25, 26, 27];
- estimate the quit rate at the end of pregnancy for women who access NHS SSS;
- using published estimates, predict the impact of the addition of TMB, EB and CFIB interventions on the quit rate of NHS SSS;
- estimate the impacts on present value of life‐time health‐care costs and benefits for both mother and her offspring; and
- estimate the cost‐effectiveness of TMB, EB and CFIB compared to NHS SSS.

Our research question and analysis plan were not pre‐registered, and hence our analysis should be considered exploratory.

### Defining the cohort

An estimated 59 066 mothers were smoking at delivery in England in 2019–20 [28]; however, only 13 799 pregnant women accessed available cessation support [29]. It was assumed that any new cessation intervention was to be delivered additive to NHS SSS, and hence would only be available to those who accessed standard cessation support. Accordingly, ESIP was programmed with a virtual cohort of 13 799 with a mean age of 31 years, corresponding to the average age of women giving birth in 2019–20 [30].

### Defining, costing and estimating the quit rate of usual care for smoking cessation in pregnancy

Three surveys of English NHS Stop Smoking Services (NHS SSS) found that nearly all services which responded provided behavioural support and nicotine replacement therapy (NRT) [31, 32, 33]. Other interventions were rarely provided; only 14% reported using incentives and 11% reported using e‐cigarettes [33]. These were excluded from our definition of usual care. Referral to NHS SSS can be made at any time during pregnancy, including the booking appointment, which usually occurs at or before 10 weeks’ gestation. Therefore, ESIP included all within‐pregnancy morbidities, including fetal loss occurring before 24 weeks (e.g. miscarriage).

From NHS SSS statistics in 2019–20, 3354 of 13 799 women who accessed NHS SSS were confirmed as successful quitters by carbon monoxide (CO) validation at 4 weeks post quit date [29], an estimated proportion of 24.31% [95% confidence interval (CI) = 23.60–25.03%, Jeffreys’ interval for 95% CI] [34]. With most quit attempts made early in pregnancy, a re‐analysis of UK‐based studies that were part of a systematic review estimated that 53.15% (95% CI = 49.83–56.47%) of women who achieved abstinence by 4 weeks post quit date would re‐start smoking by the end of pregnancy (see Supporting information, S1) [25]. Hence, we reduced the NHS SSS 4 weeks post quit date cessation rate to 11%. We identified an RCT of NRT with behavioural support as indicative of our definition of usual care to estimate the costs of delivering NHS SSS cessation care [35]. Estimated quit rates at delivery and costs for NHS SSS are given in Supporting information, S2.

### Cessation interventions: description, quit rate and costs

We estimated the impact of adding three smoking cessation interventions to routine NHS SSS care compared with routine care alone. Interventions were selected for being different to currently delivered cessation care, but also feasible for delivery in addition to this. Furthermore, all interventions had RCTs which tested usual care versus usual care plus the tested intervention, providing data ideally suited to our modelling needs. The interventions were:

- a low‐cost, tailored, self‐help, interactive smoking cessation TMB intervention delivered over a 12‐week period to women (MiQuit) [20, 21];
- an EB intervention whereby women received 14 physical activity sessions consisting of supervised treadmill exercise and physical activity consultations, plus six weekly behavioural support sessions (LEAP) [24]; and
- a CFIB intervention, whereby women received up to £400 in shopping vouchers (£50 for setting quit date, £50 if biochemically confirmed continued abstinent at 4 weeks post quit date, £100 for biochemically validated continued abstinence at 12 weeks post quit date and £200 for validated abstinence at 34–38 weeks’ gestation (CPIT) [22, 23].

To estimate quit rates at delivery which might occur when each of the three interventions were added to routine NHS SSS care, we applied the reported odds ratios or relative risks for stopping smoking by delivery to the NHS SSS quit rate (see Supporting information, S2). For interventions’ costs, study publications reported discrete intervention costs separate to those of usual care [20, 21, 22, 23, 24]. Hence, the total cost for these interventions is the summation of the study reported cost and per‐participant NHS SSS service cost. For all interventions, only the costs associated with delivering the intervention to pregnant women were included as health‐care costs associated with treating smoking‐related illness in pregnancy or childbirth is inbuilt to the ESIP model. All costs were inflated to 2019–20 prices using the appropriate inflation index from the Hospital Pay and Prices Index [36]. Total intervention costs are reported in Supporting information, S2.

### Other model inputs

Other ESIP inputs, e.g. prevalence and health‐care costs associated with possible within‐pregnancy complications and life‐time chronic diseases were left unchanged as, also, were utility weights for life‐years to estimate QALYs [25, 27].

### Measures of cost‐effectiveness

The primary measure of cost‐effectiveness was the incremental cost‐effectiveness ratio (ICER) per additional QALY for combined maternal and child outcomes with a ‘life‐time’ horizon. Secondary measures included ICERs per additional QALY for the mother and child separately, per‐additional life year and per‐additional quitter. For the end of pregnancy time horizon, ESIP also generated numbers of selected maternal complications (placenta abruption, placenta previa and pre‐eclampsia), fetal loss (miscarriage, ectopic pregnancies and stillbirths), premature births and low birth weight infants. Return on investment (ROI) figures were also estimated for mothers and infants, both separately and combined, at the life‐time horizon. ROIs are estimated by dividing incremental health‐care savings minus incremental intervention cost by the incremental intervention cost [37].

### Analytical strategy

The evaluation was conducted from an NHS and Personal Social Services perspective, with all costs and outcomes discounted at 3.5% per annum as per UK guidelines [16], using a four‐way incremental analysis as applied in multiple technology appraisals [16, 38]. We started with NHS SSS stop smoking care as the baseline, and then added the three interventions to NHS SSS separately, with ESIP estimating the associated costs and health outcomes. After NHS SSS alone, the three interventions were ranked by total combined cost for mother and offspring throughout the life‐time, from cheapest to most expensive. The interventions were evaluated in order of cost, starting with the cheapest of the three interventions compared to NHS SSS alone, followed by the second cheapest intervention compared with the cheapest, and so on. Any interventions that were dominated (i.e. more expensive and/or less effective than what they are being compared to), or extendedly dominated (when the cost‐effectiveness measure is higher than the next more effective intervention) [39], were removed from the analysis and the measures of cost‐effectiveness were re‐calculated.

### Probabilistic sensitivity analyses

To estimate the impact of uncertainty [40], ESIP conducts a probabilistic sensitivity analysis (PSA), whereby all inputs are allowed to vary within specified settings [41]. Because there are many inputs, ESIP performs 10 000 Monte Carlo simulations [41, 42], estimating pairwise incremental costs and benefits. Output from the PSA is demonstrated by incremental cost‐effectiveness plane scatterplots and cost‐effectiveness acceptability curves (CEACs) [43].

### Scenario analysis

There is evidence that reduced smoking in pregnancy decreases fetal loss [5, 6] and this is factored into ESIP, whereby interventions which reduce smoking rates generate higher estimates for numbers of live births, and so some infants who would otherwise have died during pregnancy are awarded life‐years, representing economic gain. This can be considered controversial [44], therefore a scenario analysis in which all fetal loss was removed from ESIP was conducted. We reduced the cohort of 13 799 to 12 413, based upon the number of women expected to suffer a fetal loss under current standard care by ESIP. We then re‐evaluated the interventions, including performing a second PSA.

A further series of one‐way scenario analyses were conducted whereby key model inputs were varied between ×5 and ×2 their initial value. Details of these scenario analyses can be found in Supporting information, S3.

### Data access

Data used for describing the cohort, intervention and comparator quit rates and costs are from nationally publicized data sets or previously published literature, all details of which are cited in the main text. The ESIP model is freely available to interested parties, and can be found at: https://www.nottingham.ac.uk/research/groups/tobaccoandalcohol/smoking-in-pregnancy/esip/index.aspx. All data regarding ESIP inputs can be made available on reasonable request to the corresponding author or through the ESIP website.

## RESULTS

### Base‐case incremental analysis

Deterministic incremental results can be found in Table 1, while expected outcomes can be found in Supporting information, S4. The addition of any of the three interventions to NHS SSS were found to dominate NHS SSS alone (reduced health‐care costs and improved health outcomes). However, the addition of CFIB was found to also dominate both TMB and EB, suggesting that CFIB falls below commonly accepted UK cost‐effectiveness thresholds [45]. ROIs suggested that the NHS SSS plus CFIB offered a return of £0.61 in health‐care cost reductions for every pound spent on delivering the intervention. Although the addition of TMB and EB were estimated to offer positive return on investment when compared with CFIB, this was due to the costs of delivering these interventions being much lower, and not to any greater health‐care savings or improved health outcomes generated by them.

**TABLE 1:** Base‐case (deterministic) findings for incremental outcomes and incremental cost‐effectiveness ratios (ICERs) for National Health Service Stop Smoking Services (NHS SSS) plus cessation‐contingent financial incentive (CFIB), exercise (EB) and text message (TMB)‐based interventions, versus NHS SSS alone, for a cohort of 13 799 pregnant women (see Supporting information, S2 for expected outcomes)

Intervention	NHS SSS + CFIB		NHS SSS + TMB				NHS SSS + EB			
Comparator	NHS SSS		NHS SSS + CFIB		NHS SSS		NHS SSS + CFIB		NHS SSS	
Outcome and time horizon	Incremental	ICER (£)	Incremental	ICER (£)	Incremental	ICER (£)	Incremental	ICER (£)	Incremental	ICER (£)
Expected cost (£)										
Mother only	56		−82		−25		−49		7	
Offspring only	11		−48		−37		−13		−1	
Combined	−106		40		−66		82		−24	
Quit rate at end of pregnancy (%)										
Mother only	18	305	−14	592	5	−544	−15	330	4	201
Combined		−575		−288		−1423		−550		−678
Life‐years gained										
Mother only	0.02	2924	−0.01	5675	0.00	−5218	−0.02	3161	0.00	1930
Offspring only	0.16	72	−0.12	403	0.04	−910	−0.13	100	0.03	−48
Combined	0.18	−593	−0.13	−297	0.05	−1469	−0.14	−567	0.03	−700
QALYs gained										
Mother only	0.05	1165	−0.04	2261	0.01	−2079	−0.04	1260	0.01	769
Offspring only	0.17	67	−0.13	375	0.04	−845	−0.14	93	0.03	−45
Combined	0.22	−482	−0.16	−241	0.06	−1193	−0.18	−461	0.04	−569
Return on investment (£)		1		0		15		1		1

QALYs = quality‐adjusted life‐years.

CI = confidence interval; QALYs = quality‐adjusted life‐years.

### Probabilistic incremental analysis

**FIGURE 1:**
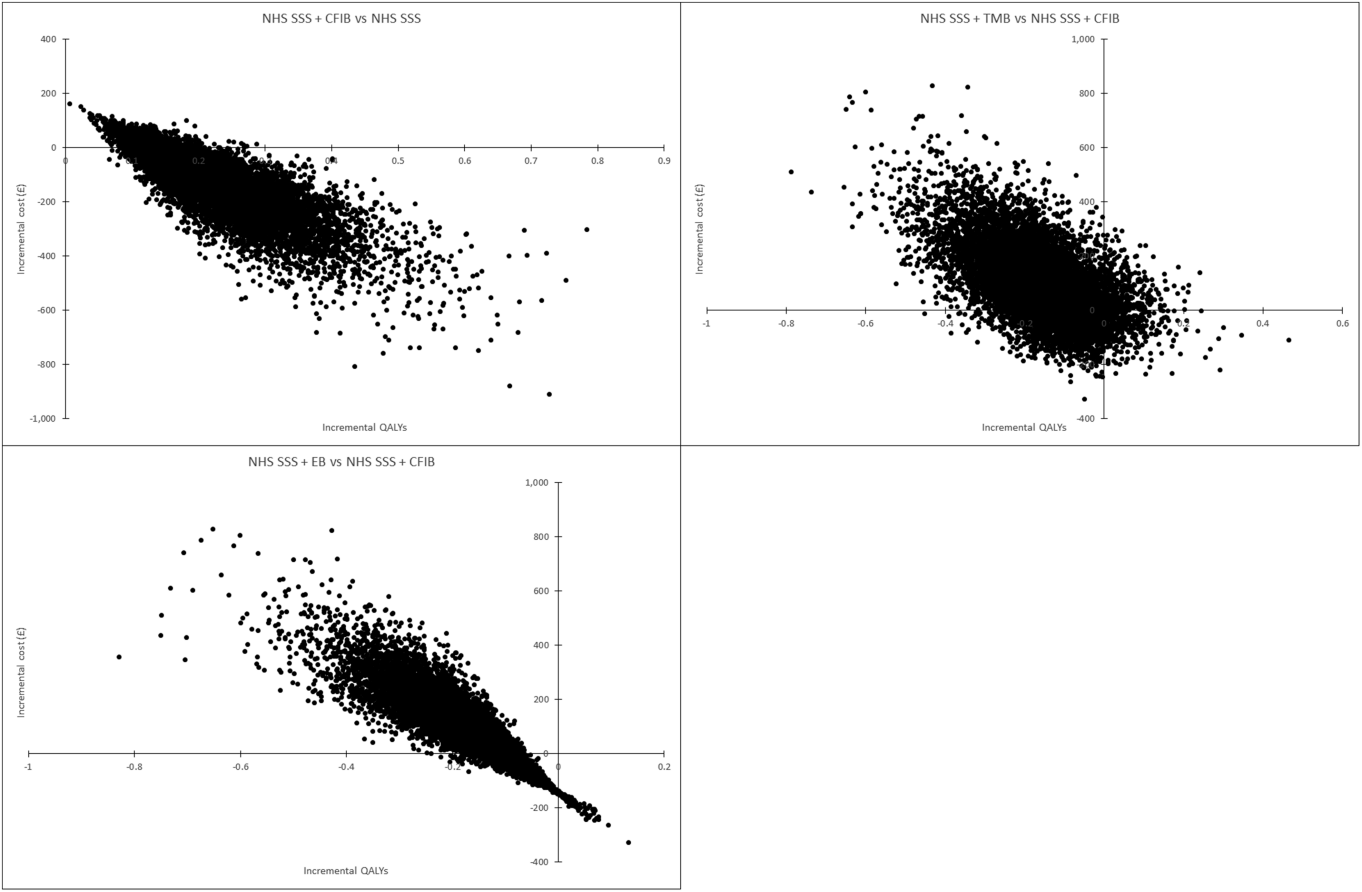
Scatterplot of incremental costs plotted against incremental quality‐adjusted life‐years (QALYs) for National Health Service Stop Smoking Services (NHS SSS) plus cessation‐contingent financial incentive (CFIB) versus NHS SSS, NHS SSS plus text message (TMB) and NHS SSS plus exercise (EB) versus NHS SSS plus CFIB for combined mother and offspring over the life‐time and adulthood‐time horizon

**FIGURE 2:**
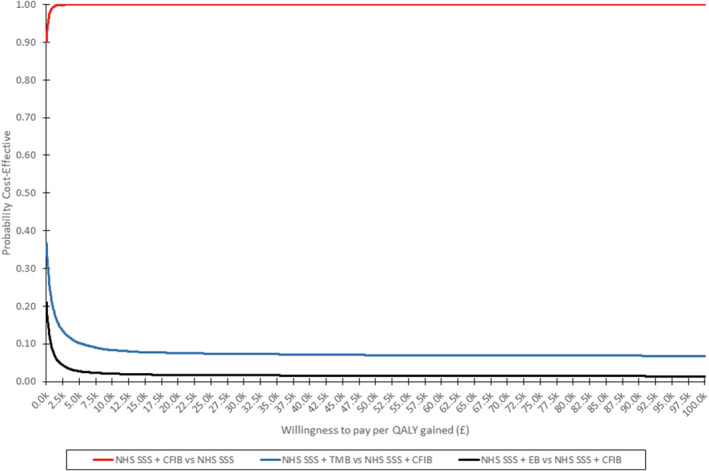
Cost‐effectiveness acceptability curve for National Health Service Stop Smoking Services (NHS SSS) plus cessation‐contingent financial incentive (CFIB) versus NHS SSS and NHS SSS plus text message (TMB) and NHS SSS plus exercise (EB) versus NHS SSS plus CFIB

Mean values and 95% CI for expected costs, life‐years and QALYs estimated for NHS SSS and the three interventions in the PSA can be found in Supporting information, S5, while the resulting incremental analysis can be found in Table 2. Only CFIB was found to be significantly better than NHS SSS alone, with both TMB and EB having iterations where expected incremental QALYs compared to NHS SSS alone were negative. Although CFIB appeared better than TMB and EB the result was not statistically significant, suggesting that there were iterations in the PSA where either or both these interventions were preferable to the CFIB. As can be seen in the scatterplots (Figure 1), in most iterations both TMB and EB lead to either lower health gains and/or greater total health‐care costs compared to CFIB. This is also reflected in the CEAC (Figure 2), where CFIB was found to have at least a 90% chance of being preferred to NHS SSS alone, while TMB and EB had at most a 38 and 21% chance of being preferred to CFIB. ROIs suggested that no intervention had a significant return compared to NHS SSS alone, although the 95% CI for CFIB was the only ROI which did not go below zero (see Supporting information, S6 for incremental values).

**TABLE 2:** Probabilistic sensitivity analysis findings for incremental outcomes and incremental cost‐effectiveness ratios (ICERs) for National Health Service Stop Smoking Services (NHS SSS) cessation‐contingent financial incentive (CFIB), exercise (EB) and text message (TMB)‐based interventions, versus NHS SSS alone, for a cohort of 13 799 pregnant women (see Supporting information, S3 for expected outcomes)

Intervention	NHS SSS + CFIB			NHS SSS + TMB						NHS SSS + EB					
Comparator	NHS SSS			NHS SSS + CFIB			NHS SSS			NHS SSS + CFIB			NHS SSS		
Outcome and time horizon	Mean	95% CI		Mean	95% CI		Mean	95% CI		Mean	95% CI		Mean	95% CI	
Incremental expected cost (£) for life‐time															
Mother only	42	−81	122	−75	−202	64	−33	−141	32	−39	−133	88	3	−57	46
Offspring only	−8	−187	108	−40	−215	158	−47	−201	43	1	−130	186	−7	−90	52
Combined	−140	−420	48	54	−248	379	−85	−336	70	106	−118	400	−34	−171	68
Incremental quit rate at the end of pregnancy (%)															
Mother only	19.15	8.12	34.25	−13.68	−31.94	4.66	5.46	−3.96	19.94	−15.26	−31.60	−1.56	3.89	−2.36	12.00
ICER mother (£)	354	−255	1467	2396	−8151	9386	168	−1742	310	564	−373	4470	−1465	−9130	9297
ICER combined (£)	−593	−1356	567	1449	−9138	8434	−780	−2800	−579	−383	−1484	3577	−2413	−10 170	8349
Incremental life‐years gained for life‐time															
Mother only	0.02	0.01	0.04	−0.01	−0.04	0.00	0.01	0.00	0.02	−0.02	−0.04	0.00	0.00	0.00	0.01
Offspring only	0.17	0.07	0.32	−0.12	−0.30	0.04	0.05	−0.03	0.18	−0.13	−0.29	−0.01	0.03	−0.02	0.11
Combined	0.19	0.07	0.36	−0.13	−0.33	0.05	0.05	−0.04	0.20	−0.15	−0.33	−0.02	0.04	−0.02	0.12
ICER mother (£)	3583	−2327	15 160	29 610	−79 152	91 598	3465	−17 787	3222	5375	−3484	45 765	−16 105	−87 998	90 658
ICER offspring (£)	104	−773	1441	4291	−9812	10 462	−219	−2574	72	307	−949	4991	−2624	−11 281	10 758
ICER combined (£)	−626	−1569	581	2951	−9515	8676	−885	−3186	−563	−441	−1765	3686	−3021	−10 713	8866
Incremental QALYs gained for life‐time															
Mother only	0.05	0.02	0.11	−0.04	−0.10	0.01	0.01	−0.01	0.06	−0.04	−0.10	0.00	0.01	−0.01	0.04
Offspring only	0.18	0.07	0.35	−0.13	−0.32	0.04	0.05	−0.04	0.19	−0.14	−0.32	−0.01	0.04	−0.02	0.12
Combined	0.23	0.09	0.44	−0.17	−0.41	0.06	0.07	−0.05	0.25	−0.18	−0.40	−0.02	0.05	−0.03	0.15
ICER mother (£)	1486	−972	6518	11 177	−32 292	38 213	−419	−8207	1216	1644	−1492	18 841	−6464	−37 225	38 040
ICER offspring (£)	98	−721	1332	4258	−9323	9909	−170	−2442	69	293	−886	4607	−2408	−10 681	9868
ICER combined (£)	−506	−1247	473	2398	−7806	7161	−751	−2558	−450	−374	−1405	2983	−2374	−8754	6917
Return on investment (£)	2	1	3	1	0	3	21	−15	81	2	0	4	2	−1	7

### Scenario analysis

After removing fetal loss, the overall findings of cost‐effectiveness remained similar to the base case, and hence are not reproduced in detail here (see Supporting information, S7–S11). CFIB remained the preferred intervention in both deterministic and probabilistic sensitivity analyses. There were no changes in the overall ranking of the interventions; however, the value for money associated with CFIB increased while the value for money TMB and EB versus CFIB decreased. This was because ESIP estimated an increase in incremental health‐care savings for CFIB versus NHS SSS alone, and an increase in incremental expenditure for TMB and EB versus CFIB. This reduced the maximum chance that TMB and EB were considered cost‐effective compared to CFIB to approximately 28 and 12%, respectively; meanwhile the minimum chance of CFIB cost‐effectiveness versus NHS SSS alone increased to 98%. The other difference in ESIP outputs for the scenario analysis was that all three interventions were estimated to increase the number of maternal complications rather than the decrease as estimated in the base case. This is due to ESIP estimating an increase in pregnancies with pre‐eclampsia, as within‐pregnancy smoking has been estimated to reduce the risk of pre‐eclampsia [6].

Results of the chosen scenario analyses can be found in Supporting information, S3. ICERs appeared to be more sensitive to changes in intervention effectiveness than to changes in per participant cost or changes in the NHS SSS quit rate. CFIB remained dominant in all but two scenarios. In one scenario it was more effective but more expensive than the next best intervention, with relatively low ICERs. In the second, CFIB was dominated by TMB only when the effectiveness of CFIB was reduced to a low value.

## DISCUSSION

We have investigated the potential cost‐effectiveness of adding three interventions for helping pregnant women to stop smoking to current UK NHS SSS. Our findings suggest that in England, the greatest benefit can be gained by implementing the CFIB intervention in addition to NHS SSS. Although NHS SSS plus TMB and EB interventions appeared to offer benefits over NHS SSS alone, these were non‐significant and overpowered by the addition of CFIB.

### Strengths

The ESIP model is peer‐reviewed and addresses many shortcomings highlighted in previous EEs of smoking cessation interventions within pregnancy [18, 25, 27]. Although the original RCTs for three interventions included EEs [21, 22, 24], those conducted for the EB and TMB had only end‐of‐pregnancy time horizons. The evaluation of the CFIB used a ‘life‐time’ horizon for analyses but was limited to the mother only, and economic impacts attributable to within‐pregnancy smoking on offspring were not considered. By incorporating the trials’ findings into the inputs of ESIP, we have predicted the economic impacts of these interventions. Furthermore, both the PSA and one‐way scenario analyses demonstrate robustness that the addition of CFIB to NHS SSS is dominant under decision uncertainty, even after the removal of any life‐years gained from fetal loss [46].

### Limitations

When developing the cohort for ESIP, only two covariates are required: mean age and year of birth. Other covariates that may be of relevance are omitted, e.g. socio‐economic status may be influential in the circumstance of the financial rewards participants receive under CFIB. It may be possible to pre‐define a profile cohort of women; however, this would require the alteration of several ESIP model inputs.

Fetal loss is incorporated into ESIP, but English national data sources do not report information on pregnancy outcomes among women who accessed NHS SSS. Therefore, it is difficult to know whether women who suffered fetal loss were included in the national data [29]. This uncertainty could impact the NHS SSS quit rate, although in which direction is unclear. However, the scenario analysis demonstrated robustness of cost‐effectiveness after the removal of fetal loss.

ESIP assumes that the return to smoking after pregnancy is the same across all interventions [47]; however, it is possible that post‐birth returning‐to‐smoking rates vary with the type of intervention used to achieve within‐pregnancy abstinence. Many trials testing within‐pregnancy cessation interventions do not collect data on post‐birth return to smoking. None were collected for TMB [21]. However, both the trials for EB and CFIB collected data at 6 months postpartum [23, 24]; the only significant difference between control and intervention groups was in the trial for the CFIB [22, 23]. Return to smoking postpartum is important, as smoking behaviour impacts upon both the health of the mother and the child [48], as well as increasing the child's risk of becoming a smoker in the future [49]. If interventions have different return‐to‐smoking rates, this could impact upon cost‐effectiveness. For example, should CFIB have a higher rate of post‐birth smoking return than other interventions, this could reduce the cost‐effectiveness of CFIB despite the higher quit rate within‐pregnancy, and result in another intervention being preferred. However, the CFIB RCT found that 6 months postpartum cessation rates were still approximately three times higher in women randomized to CFIB [22, 23], whereas a trial testing routine care found no difference in cessation rates at this point [50]. Hence, there is no evidence for that postpartum re‐start following CFIB would be higher than following cessation with other interventions.

### In context with the literature

Previous work used to inform NICE guideline development found a similar result [51, 52, 53]. Taylor's work modelled the impact of several cessation interventions on a national scale [52] and drew a similar conclusion that ‘reward’‐based interventions appeared to dominate all other interventions, including pharmacotherapy, cognitive behaviour strategies and feedback‐based interventions. Mallender et al. found that intensive behavioural interventions appeared dominant; however, the three conditional, incentive‐based interventions evaluated were still cost‐effective as ICERs per QALY gained were relatively small (£412, £788 and £388 in 2011 prices) [53]. Therefore, our finding of CFIB being value for money is not unsurprising, and is probably a robust result.

The original evaluation of CFIB modelled outcomes and health‐care costs of women up to age 100 years [22, 23]. The findings suggested that there was a chance that CFIB was not considered cost‐effective, with a scatterplot that covered all four quadrants of the cost‐effectiveness plane. ESIP's replication was different with a replica scatterplot, found in Supporting information, S12, showing none of the iterations to the left of the vertical axis. The difference in the two findings may be due to the small cohort used within the trial and some of the key differences between the two approaches, such as the exclusion of impacts on the offspring and how post‐pregnancy smoking behaviour was evaluated.

### Application and implication for policy

Compared with NHS SSS alone, the addition of TMB, EB or CFIB appears cost‐effective, highlighting the benefits gained from further encouragement given to pregnant smokers to quit. Furthermore, within‐pregnancy cessation interventions can easily return their additional costs through health‐care savings. The largest returns were associated with CFIB despite TMB and EB offering some benefits. Since we started this analysis, NICE have updated their guidance on managing smoking in pregnancy and now recommends that women are offered financial incentives contingent upon their abstinence on smoking [51]. Our work further validates this decision. Despite findings from our analyses only directly translating to the UK NHS context, it is likely that comparable benefits from CFIB would occur if incorporated into similar health systems.

However, any new policy should consider that the addition of CFIB is not without ethical issues, as the authors of the original RCT highlighted [23]. Concerns expressed are that it encourages women to game the intervention [54], and can be seen as unfair to non‐smoking pregnant women [55]. One reason for using financial incentives is they have ‘appeal’ to women whose income is restricted [54], in particular those of lower socio‐economic status or very young mothers. It has been demonstrated that the burden of smoking is higher in more socio‐economically disadvantaged groups [56], therefore it is prudent to address this issue. It may be that the addition of CFIB to NHS SSS would improve the reach of such services, given that in 2019–20 only 13 799 women set a quit date while 59 066 women were estimated to be smoking at delivery during the same period [29]. This suggests that there are still 45 267 women who do not access NHS SSS. Increased engagement with services would obviously lead to health benefits from cessation, although evidence suggests that differences exist between women who engage with NHS SSS to those who do not, e.g. they are less socio‐economically deprived and more likely to be employed in a managerial or professional occupation [57]. Therefore, it could be questioned whether offering financial incentives would be of benefit to these women. Despite ethical considerations, CFIB during pregnancy appears cost‐effective, offering the largest health gains and health‐care savings compared to other available cessation interventions.

## DECLARATION OF INTERESTS

None.

## AUTHOR CONTRIBUTIONS

**Matthew Jones:** Conceptualization; formal analysis; investigation; methodology; validation; visualization. **Murray Smith:** Formal analysis; investigation; methodology; supervision; validation; visualization. **Sarah Lewis:** Conceptualization; methodology; validation. **Steve Parrott:** Conceptualization; methodology; supervision; validation. **Tim Coleman:** Conceptualization; funding acquisition; methodology; supervision; validation; visualization.

## Supporting information


**Data S1.** Supporting information
