# Supplementary material for: Investigating the cost‐effectiveness of three cessation interventions on a national scale using the Economics of Smoking in Pregnancy (ESIP) decision analytical model
Source: Addiction. 2022 Jun 14;117(11):2907–17. doi: 10.1111/add.15968 (PMC9796630; doi:10.1111/add.15968)
Supplement: Supplementary file 1 — Data S1. Supporting information [file ADD-117-2907-s001.pdf]

Supplementary Information

Supplementary Information 1: *Meta-analysis of UK studies included in Jones et al (2016) (1) using a random effects model, with abstinence defined as women who reported biochemically validated continuous abstinence at end of pregnancy given biochemically validated abstinence at four weeks post quit date. Note: The estimated proportion of women who restart smoking is defined as one minus proportion abstinent.*

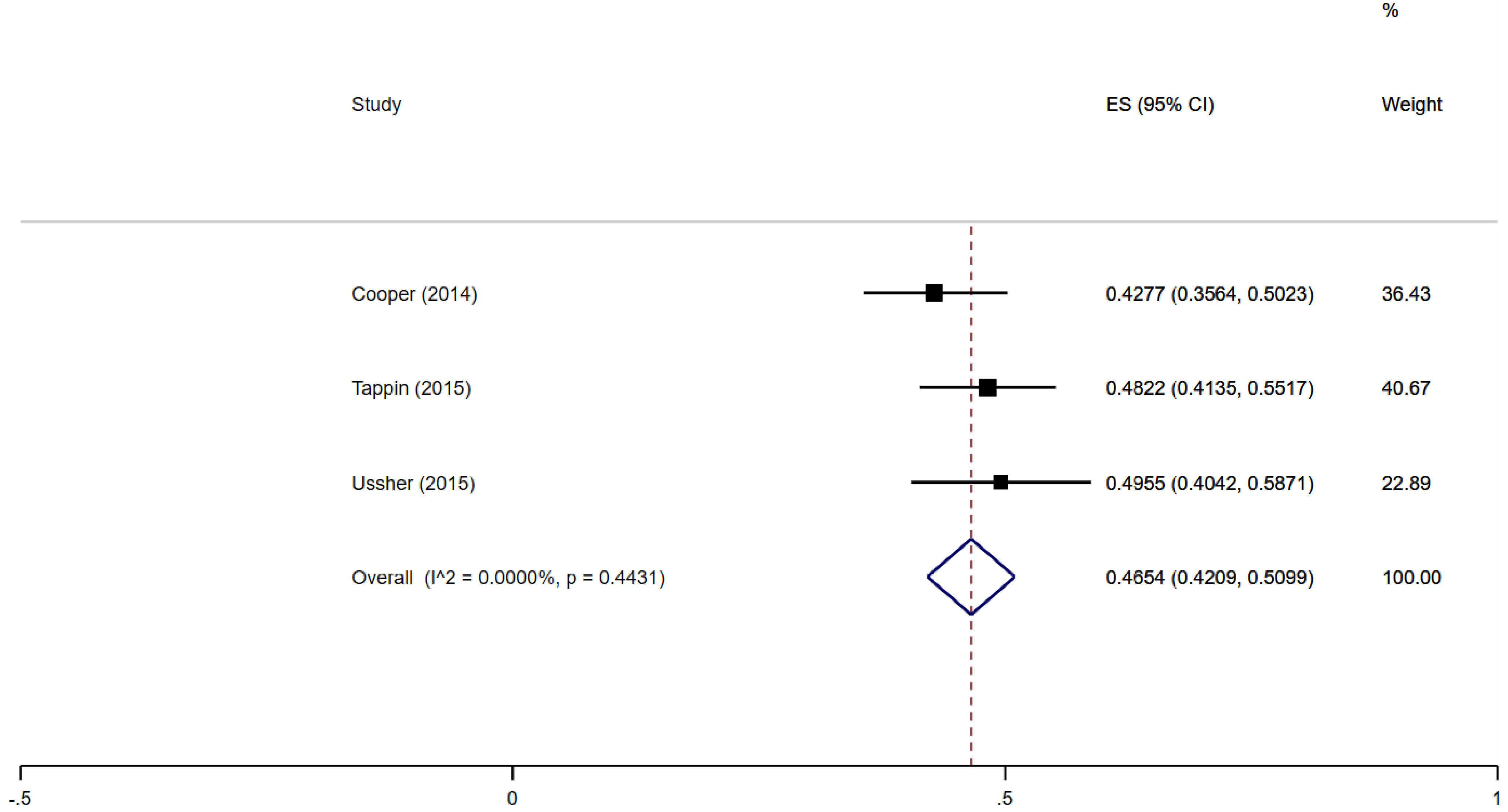

Supplementary Information 2: *Model inputs for quit rates and costs of NHS SSS and interventions as programmed into the ESIP model using study reported results.*

**Model input**	**Estimated quit rate (%) used in ESIP**			
	**Mean**	**95% CI**		**Distribution fitted**
NHS SSS 4 week post quit date quit rate (%)	24.31	23.60	25.03	Beta
Proportion of women who restart smoking between quit date and end of pregnancy (%)	46.54	42.09	50.99	Beta
Predicted NHS SSS quit rate at end of pregnancy	11.31[1]			
Odds ratio for effectiveness of NHS SSS + Text Message (2)	1.49	0.62	3.6	Log Normal
Predicted quit rate of NHS SSS + Text Message	15.97[2]			
Odds ratio for effectiveness of NHS + Exercise (3)	1.37	0.78	2.41	Log Normal
Predicted quit rate of NHS SSS + Exercise	14.88[2]			
Relative risk for effectiveness of NHS + Cessation-contingent financial incentive (4)	2.63	1.73	4.01	Log Normal
Predicted quit rate of NHS SSS + Cessation-contingent financial incentive	29.76[3]			
Cost of NHS SSS per participant	116.27	113.24	119.3	Gamma
Additional cost per participant for Text Message	4.32	3.47	5.17	Gamma
Total cost per participant for NHS SSS + Text Message	120.59[4]			
Additional cost per participant for Exercise	29.86	24.57	35.15	Gamma
Total cost per participant for NHS SSS + Exercise	146.13[4]			
Additional cost per participant for Cessation-contingent financial incentive	173.62	170.12	177.12	Gamma
Total cost per participant for NHS SSS + Cessation-contingent financial incentive	289.89[4]			

[1] Estimated by multiplying NHS SSS 4 week quit rate by proportion still abstinent at end of pregnancy

[2] Estimated by converting probability quitting of NHS SSS to odds, multiplying by the relevant odds ratio, then reconverting the value to a probability

[3] Estimated by multiplying probability quitting of NHS SSS by relative risk of intervention

[4] Estimated by summing NHS SSS cost per participant plus additional cost of intervention

Supplementary information 3: One-way scenario analyses on model inputs from SI 2, low values corresponding to 0.5x initial model input, and high values corresponding to 2x initial model input.

Model input	Low value	High value	Result of evaluation	NHS SSS + CFIB vs NHS SSS ICER (£)			NHS SSS + TMB vs NHS SSS + CFIB ICER (£)			NHS SSS + EB vs NHS SSS + CFIB ICER (£)		
				Min	Max	Range	Min	Max	Range	Min	Max	Range
NHS SSS Quit rate at end of pregnancy	NHS SSS QR: 5.66% TMB QR: 8.20% EB QR: 7.59% CFIB QR: 14.88%	NHS SSS QR: 22.63% TMB QR: 30.35% EB QR: 28.61% CFIB QR: 59.51%	At low value, NHS SSS + TMB dominated NHS SSS and NHS SSS + EB, NHS SSS + CFIB was more expensive and more effective than NHS SSS + TMB, with a ICER of £854.<br><br>At high value, NHS SSS + CFIB dominated as in main analysis	-876.50	307.50	1,184	-784.40	854.94	1,639	-881.17	382.99	1,264
Effectiveness of TMB	OR: 0.74 QR: 8.68%	OR: 2.98 QR: 27.55%	At low value, NHS SSS + TMB dominated by NHS SSS, NHS SSS + CFIB remains dominant intervention.<br><br>At high value, NHS + TMB dominates NHS SSS and NHS SSS + EB, NHS SSS + CFIB more expensive but more effective than NHS SSS + TMB, with ICER per QALY of £5,149.	-481.83	-481.83	0	-597.68	5,149.37	5,747	-461.04	-461.04	0
Effectiveness of EB	OR: 0.69 QR 8.04%	OR: 2.74 QR 25.90%	At low value, NHS SSS + CFIB dominates as per main analysis.<br><br>At high value, NHS SSS + EB dominates both NHS SSS and NHS SSS + TMB. NHS + CFIB is more expensive but more effective than NHS SSS + EB, with ICER per QALY of £1,856.	-481.83	-481.83	0	-241.34	-241.34	0	-716.21	1,855.81	2,572
Effectiveness of CFIB	RR: 1.32 QR: 14.37%	RR: 5.26 QR: 40.16%	At low value, NHS SSS + TMB is dominant intervention.<br><br>At high value, NHS SSS + CFIB is dominant intervention as per main analysis.	-766.46	3,498.61	4,265	-10,107.29	-684.22	9,423	-24,837.97	-794.35	24,044
NHS SSS Cost per participant	£58.14	£232.54	NHS SSS + CFIB dominant as in main analysis in both scenarios	-481.83	-481.83	0	-241.34	-241.34	0	-461.04	-461.04	0
Additional cost per participant of TMB	£2.16	£8.64	NHS SSS + CFIB dominant as in main analysis in both scenarios	-481.83	-481.83	0	-267.61	-228.20	39	-461.04	-461.04	0
Additional cost per participant of EB	£14.93	£59.72	NHS SSS + CFIB dominant as in main analysis in both scenarios	-481.83	-481.83	0	-241.34	-241.34	0	-629.32	-376.92	252
Additional cost per participant of CFIB	£86.81	£347.24	At low value, NHS SSS + CFIB dominant as in main analysis.<br><br>At high value, NHS SSS + TMB dominates both NHS SSS and NHS SSS + EB. NHS SSS + CFIB more expensive but more effective than NHS SSS + TMB, with ICER per QALY of £815.	-876.49	307.53	1,184	-769.39	814.80	1,584	-950.23	517.37	1,468

Supplementary Information 4: Basecase (deterministic) findings for expected and incremental outcomes for NHS SSS plus cessation-contingent financial incentive (CFIB), Exercise (EB), and Text-message (TMB) based interventions, versus NHS SSS alone, for a cohort of 13,799 pregnant women.

	**NHS SSS**	**NHS SSS + CFIB**		**NHS SSS + TMB**			**NHS SSS + EB**		
**Outcome & Time Horizon**	**Estimate**	**Estimate**	**Incremental vs NHS SSS**	**Estimate**	**Incremental vs NHS SSS + CFIB**	**Incremental vs NHS SSS**	**Estimate**	**Incremental vs NHS SSS + CFIB**	**Incremental vs NHS SSS**
End of pregnancy									
Quit rate at end of pregnancy (%)	11.31	29.76	18.44	15.97	-13.78	4.66	14.88	-14.88	3.56
Maternal complications	1,649	1,590	-59	1,634	44	-15	1,638	48	-11
Foetal loss including stillbirth	1,422	1,342	-80	1,402	60	-20	1,407	65	-16
Premature births	991	960	-31	984	23	-8	985	25	-6
Low birth weight	1,484	1,342	-142	1,448	106	-36	1,457	114	-27
Maternal lifetime (100 years of age)									
Expected cost (£)	11,200.46	11,256.66	56.20	11,175.12	-81.54	-25.34	11,207.63	-49.03	7.17
Expected life years	24.6223	24.6415	0.0192	24.6271	-0.0144	0.0049	24.6260	-0.0155	0.0037
Expected QALYs	22.3660	22.4142	0.0482	22.3782	-0.0361	0.0122	22.3753	-0.0389	0.0093
ICER per quitter gained (£)			*304.73*		*591.56*	*-543.95*		*329.53*	*201.21*
ICER per life year gained (£)			*2,923.51*		*5,675.22*	*-5,218.45*		*3,161.37*	*1,930.36*
ICER per QALY gained (£)			*1,164.76*		*2,261.08*	*-2,079.09*		*1,259.53*	*769.08*
Offspring lifetime									
Expected cost (£)	7,865.19	7,876.63	11.44	7,828.55	-48.08	-36.65	7,863.71	-12.92	-1.48
Expected life years	24.1926	24.3520	0.1595	24.2328	-0.1192	0.0403	24.2234	-0.1287	0.0308
Expected QALYs	23.6677	23.8394	0.1717	23.7110	-0.1283	0.0434	23.7008	-0.1385	0.0332
ICER per life year gained (£)			*71.70*		*403.35*	*-909.61*		*100.37*	*-48.00*
ICER per QALY gained (£)			*66.60*		*374.67*	*-844.94*		*93.23*	*-44.59*
Combined maternal and offspring									
Expected cost (£)	18,949.38	18,843.40	-105.98	18,883.08	39.67	-66.31	18,925.21	81.81	-24.17
Expected life years	48.8148	48.9936	0.1787	48.8600	-0.1336	0.0451	48.8494	-0.1442	0.0345
Expected QALYs	46.0336	46.2536	0.2200	46.0892	-0.1644	0.0556	46.0761	-0.1775	0.0425
ICER per quitter gained (£)			*-574.66*		*-287.84*	*-1,423.34*		*-549.87*	*-678.18*
ICER per life year gained (£)			*-593.00*		*-297.02*	*-1,468.76*		*-567.41*	*-699.82*
ICER per QALY gained (£)			*-481.83*		*-241.34*	*-1,193.42*		*-461.04*	*-568.63*
Return on Investment									
Expected intervention cost (£) *(Intervention - Comparator)*	116.27	289.89	173.62	120.59	-169.30	4.32	146.13	-143.76	29.86

Maternal healthcare cost (£) *(Comparator - Intervention)*	11,084.19	10,966.77	117.42	11,054.53	-87.76	29.66	11,061.50	-94.73	22.69
Offspring healthcare cost (£) *(Comparator - Intervention)*	7,748.92	7,586.74	162.18	7,707.96	-121.21	40.97	7,717.59	-130.84	31.34
Combined healthcare cost (£) *(Comparator - Intervention)*	18,833.11	18,553.51	279.60	18,762.48	-208.97	70.63	18,779.09	-225.57	54.02
Return on Investment			*0.61*		*0.23*	*15.35*		*0.57*	*0.81*

Supplementary Information 5: Probabilistic analysis: Estimated outcomes for NHS SSS alone, and NHS SSS plus cessation-contingent financial incentive (CFIB), Exercise (EB), and Text-message (TMB) based interventions, at the end of pregnancy and lifetime time horizons for mother and offspring, for a cohort of 13,799 pregnant women.

	NHS SSS			NHS SSS + CFIB			NHS SSS + TMB			NHS + EB		
**Outcome**	**Mean**	**95% CI**		**Mean**	**95% CI**		**Mean**	**95% CI**		**Mean**	**95% CI**	
**End of pregnancy**												
Quit rate at delivery (%)	11.31	10.19	12.47	29.68	25.65	34.49	16.78	7.19	31.41	15.20	8.88	23.49
Any maternal complication	1,656	1,525	1,797	1,593	1,481	1,715	1,638	1,509	1,778	1,643	1,516	1,780
Foetal loss including stillbirth	1,427	1,303	1,562	1,342	1,227	1,465	1,403	1,276	1,542	1,409	1,286	1,542
Premature birth	992	941	1,045	959	912	1,010	983	930	1,039	986	935	1,040
LBW infants	1,481	1,393	1,553	1,334	1,206	1,442	1,439	1,307	1,545	1,451	1,350	1,540
**Maternal lifetime (up to age 100 years)**												
Life Year	24.64	24.52	24.75	24.66	24.54	24.77	24.65	24.52	24.76	24.65	24.52	24.76
QALY	22.36	20.89	23.46	22.41	20.97	23.49	22.38	20.91	23.47	22.37	20.91	23.47
Cost (£)	11,713.91	10,174.17	13,512.37	11,755.81	10,220.65	13,563.84	11,680.58	10,137.61	13,491.01	11,716.99	10,178.32	13,520.84
**Offspring lifetime (up to age 100 years)**												
Life Year	24.19	23.93	24.44	24.36	24.12	24.58	24.24	23.97	24.49	24.23	23.96	24.47
QALY	23.66	22.82	24.22	23.84	23.05	24.38	23.72	22.88	24.27	23.70	22.88	24.25
Cost (£)	8,308.41	5,703.54	13,670.28	8,300.53	5,686.54	13,712.09	8,260.96	5,656.05	13,612.34	8,301.56	5,692.85	13,668.57
**Combined maternal and infant lifetime (up to age 100 years)**												
Life Year	48.83	48.53	49.11	49.02	48.74	49.28	48.89	48.58	49.17	48.87	48.58	49.15
QALY	46.03	44.02	47.58	46.26	44.28	47.78	46.09	44.08	47.65	46.07	44.07	47.63
Cost (£)	19,906.05	16,606.97	25,465.02	19,766.48	16,448.27	25,415.85	19,820.96	16,523.80	25,371.37	19,872.41	16,572.24	25,419.52
**Return on Investment**												
Expected intervention cost (£)	116.27	113.25	119.31	289.86	285.20	294.53	120.58	117.43	123.73	146.14	140.18	152.52
Maternal healthcare cost (£)	11,597.65	10,057.11	13,398.38	11,465.95	9,931.85	13,274.97	11,560.00	10,017.60	13,372.85	11,570.85	10,032.97	13,378.58
Offspring healthcare cost (£)	8,192.14	5,587.61	13,553.85	8,010.67	5,396.35	13,420.97	8,140.37	5,534.83	13,491.92	8,155.42	5,547.11	13,526.53
Combined healthcare cost (£)	19,789.79	16,489.77	25,346.76	19,476.62	16,159.71	25,128.86	19,700.37	16,402.72	25,248.04	19,726.27	16,424.25	25,269.55

Supplementary Information 6: Probabilistic Analysis: Incremental return on investment for NHS SSS plus cessation-contingent financial incentive (CFIB) verses NHS SSS alone, Exercise (EB), and Text-message (TMB) based interventions versus NHS SSS + CFIB.

	**NHS SSS + CFIB vs NHS SSS**			**NHS SSS + TMB vs NHS SSS + CFIB**			**NHS SSS + TMB vs NHS SSS**			**NHS SSS + EB vs NHS SSS + CFIB**			**NHS SSS + EB vs NHS SSS**		
**Outcome & Time Horizon**	**Mean**	**95% CI**		**Mean**	**95% CI**		**Mean**	**95% CI**		**Mean**	**95% CI**		**Mean**	**95% CI**	
**Return on Investment**															
Expected intervention cost (£) *(Intervention - Comparator)*	173.60	170.03	177.08	-169.28	-172.87	-165.71	4.32	3.52	5.22	-143.72	-149.85	-137.09	29.88	24.75	35.54
Maternal healthcare cost (£) *(Comparator - Intervention)*	131.70	51.79	253.84	-94.05	-233.65	32.62	37.65	-27.74	145.24	-104.90	-230.91	-10.87	26.80	-16.02	87.03
Offspring healthcare cost (£) *(Comparator - Intervention)*	181.47	65.69	360.46	-129.70	-327.10	44.98	51.77	-38.74	205.08	-144.75	-328.77	-14.15	36.72	-22.20	119.50
Combined healthcare cost (£) *(Comparator - Intervention)*	313.17	125.28	592.87	-223.75	-548.14	78.30	89.42	-65.95	340.64	-249.65	-542.88	-26.36	63.52	-38.46	201.35

Supplementary Information 7: Deterministic findings for the scenario analysis of no foetal loss: Expected and incremental outcomes for NHS SSS plus cessation-contingent financial incentive (CFIB), Exercise (EB), and Text-message (TMB) based interventions

	NHS SSS	NHS SSS + CFIB		NHS SSS + TMB			NHS SSS + EB		
**Outcome & Time Horizon**	**Estimate**	**Estimate**	**Incremental vs NHS SSS**	**Estimate**	**Incremental vs NHS SSS + CFIB**	**Incremental vs NHS SSS**	**Estimate**	**Incremental vs NHS SSS + CFIB**	**Incremental vs NHS SSS**
End of pregnancy									
Quit rate at end of pregnancy (%)	11.31	29.76	18.44	15.97	-13.78	4.66	14.88	-14.88	3.56
Maternal complications	328	337	9	330	-7	2	329	-7	2
Premature births	984	948	-36	975	27	-9	977	29	-7
Low birth weight	1,474	1,329	-146	1,437	109	-37	1,446	117	-28
Maternal lifetime (100 years of age)									
Expected cost (£)	11,447.76	11,490.88	43.12	11,419.11	-71.76	-28.64	11,452.40	-38.48	4.64
Expected life years	24.6707	24.6873	0.0166	24.6749	-0.0124	0.0042	24.6739	-0.0134	0.0032
Expected QALYs	22.4160	22.4616	0.0456	22.4275	-0.0341	0.0115	22.4248	-0.0368	0.0088
ICER per quitter gained (£)			233.81		520.64	-614.87		258.60	130.29
ICER per life year gained (£)			2,596.30		5,781.29	-6,827.67		2,871.62	1,446.77
ICER per QALY gained (£)			944.62		2,103.43	-2,484.13		1,044.79	526.38
Offspring lifetime									
Expected cost (£)	8,831.82	8,766.41	-65.41	8,775.76	9.35	-56.06	8,815.49	49.08	-16.33
Expected life years	26.9724	26.9750	0.0025	26.9731	-0.0019	0.0006	26.9729	-0.0020	0.0005
Expected QALYs	26.3867	26.4062	0.0195	26.3916	-0.0146	0.0049	26.3905	-0.0157	0.0038
ICER per life year gained (£)			-25,985.64		-4,969.67	-88,169.20		-24,169.01	-33,570.78
ICER per QALY gained (£)			-3,357.99		-642.21	-11,393.67		-3,123.24	-4,338.18
Combined maternal and offspring									
Expected cost (£)	20,163.30	19,967.40	-195.90	20,074.28	106.88	-89.02	20,121.76	154.36	-41.54
Expected life years	51.6432	51.6623	0.0191	51.6480	-0.0143	0.0048	51.6468	-0.0154	0.0037
Expected QALYs	48.8027	48.8678	0.0651	48.8192	-0.0487	0.0165	48.8153	-0.0525	0.0126
ICER per quitter gained (£)			-1,062.24		-775.42	-1,910.92		-1,037.45	-1,165.77
ICER per life year gained (£)			-10,243.10		-7,477.28	-18,426.83		-10,004.02	-11,241.35
ICER per QALY gained (£)			-3,008.06		-2,195.83	-5,411.35		-2,937.85	-3,301.22
Return on Investment									
Expected intervention cost (£) *(Intervention - Comparator)*	116.27	289.89	173.62	120.59	-169.30	4.32	146.13	-143.76	29.86
Maternal healthcare cost (£) *(Comparator - Intervention)*	11,331.49	11,200.99	130.50	11,298.52	-97.53	32.96	11,306.27	-105.28	25.21

Offspring healthcare cost (£) *(Comparator - Intervention)*	8,715.55	8,476.52	239.02	8,655.17	-178.65	60.38	8,669.36	-192.84	46.18
Combined healthcare cost (£) *(Comparator - Intervention)*	20,047.03	19,677.51	369.52	19,953.69	-276.18	93.34	19,975.63	-298.12	71.40
Return on Investment			1.13		0.63	20.61		1.07	1.39

Supplementary Information 8: Probabilistic analysis for the scenario analysis of no foetal loss: Estimated outcomes for NHS SSS alone, NHS SSS plus cessation-contingent financial incentive (CFIB), Exercise (EB), and Text-message (TMB) based interventions, at the end of pregnancy and lifetime time horizons for mother and offspring

	NHS SSS			NHS SSS + CFIB			NHS SSS + TMB			NHS SSS + EB		
**Outcome & Time Horizon**	**Mean**	**95% CI**		**Mean**	**95% CI**		**Mean**	**95% CI**		**Mean**	**95% CI**	
**End of pregnancy**												
Quit rate at delivery (%)	11.31	10.19	12.47	29.68	25.65	34.49	16.78	7.19	31.41	15.20	8.88	23.49
Any maternal complication	331	284	385	339	301	383	333	288	385	332	288	385
Premature birth	985	935	1036	947	898	998	974	920	1029	977	927	1030
LBW infants	1471	1385	1541	1321	1189	1430	1429	1295	1536	1441	1342	1529
**Maternal lifetime (up to age 100 years)**												
Life Year	24.69	24.56	24.80	24.71	24.59	24.81	24.70	24.57	24.80	24.69	24.57	24.80
QALY	22.41	20.94	23.50	22.46	21.01	23.54	22.43	20.96	23.51	22.42	20.96	23.51
Cost (£)	11,961.86	10,334.23	13,913.19	11,989.97	10,376.21	13,932.86	11,924.60	10,295.58	13,883.92	11,962.11	10,341.14	13,917.61
**Offspring lifetime (up to age 100 years)**												
Life Year	26.98	26.95	27.00	26.98	26.96	27.01	26.98	26.95	27.01	26.98	26.95	27.01
QALY	26.39	25.49	26.93	26.41	25.57	26.94	26.40	25.50	26.93	26.40	25.51	26.93
Cost (£)	9,333.09	6,411.30	15,286.29	9,240.02	6,321.76	15,195.71	9,261.30	6,339.83	15,197.79	9,308.84	6,383.14	15,266.46
**Combined maternal and infant lifetime (up to age 100 years)**												
Life Year	51.67	51.53	51.80	51.69	51.55	51.81	51.68	51.53	51.80	51.67	51.53	51.80
QALY	48.80	46.75	50.38	48.87	46.85	50.42	48.82	46.77	50.39	48.82	46.76	50.39
Cost (£)	21,178.68	17,541.57	27,358.63	20,940.13	17,308.81	27,210.70	21,065.31	17,420.28	27,256.85	21,124.80	17,487.47	27,255.67
**Return on Investment**												
Expected intervention cost (£)	116.27	113.25	119.31	289.86	285.20	294.53	120.58	117.43	123.73	146.14	140.18	152.52
Maternal healthcare cost (£)	11,845.59	10,216.60	13,797.25	11,700.11	10,083.92	13,641.49	11,804.01	10,173.94	13,762.42	11,815.96	10,194.33	13,773.78
Offspring healthcare cost (£)	9,216.83	6,295.13	15,170.94	8,950.16	6,033.92	14,906.21	9,140.71	6,218.11	15,079.02	9,162.69	6,236.89	15,118.33
Combined healthcare cost (£)	21,062.42	17,424.42	27,241.81	20,650.27	17,018.71	26,921.64	20,944.72	17,298.98	27,135.65	20,978.66	17,341.38	27,109.60

Supplementary Information 9: Probabilistic analysis for the scenario analysis of no foetal loss: Incremental expected costs, life years, and QALYs for NHS SSS plus cessation-contingent financial incentive (CFIB) versus NHS SSS alone, Exercise (EB), and Text-message (TMB) based interventions verses both NHS SSS + CFIB and NHS SSS alone.

Outcome & Time Horizon	NHS SSS + CFIB vs NHS SSS			NHS SSS + TMB vs NHS SSS + CFIB			NHS SSS + TMB vs NHS SSS			NHS SSS + EB vs NHS SSS + CFIB			NHS SSS + EB vs NHS SSS		
	**Mean**	**95% CI**		**Mean**	**95% CI**		**Mean**	**95% CI**		**Mean**	**95% CI**		**Mean**	**95% CI**	
**Incremental expected cost (£)**															
Mother lifetime	28.12	-102.99	115.79	-65.38	-205.92	85.13	-37.26	-155.00	35.14	-27.87	-131.35	108.50	0.25	-66.28	48.02
Offspring lifetime	-93.07	-333.99	65.42	21.27	-236.59	296.16	-71.80	-288.49	60.93	68.81	-121.05	318.68	-24.25	-142.20	63.11
Combined lifetime	-238.55	-591.49	3.00	125.17	-272.70	540.88	-113.37	-441.16	91.30	184.67	-108.09	560.89	-53.88	-233.29	80.08
**Incremental expected quitters**															
Mother lifetime (%)	0.1915	0.0812	0.3425	-0.1368	-0.3194	0.0466	0.0546	-0.0396	0.1994	-0.1526	-0.3160	-0.0156	0.0389	-0.0236	0.1200
ICER per quitter gained (mother only) (£)	281.92	-321.00	1,394.10	2,324.04	-8,245.11	9,313.12	95.63	-1,815.94	255.16	492.50	-443.56	4,424.73	-1,537.29	-9,234.72	9,237.21
ICER per quitter gained (combined) (£)	-1,110.48	-1,895.41	35.38	931.64	-9,669.69	7,922.95	-1,296.77	-3,328.38	-1,101.16	-899.90	-2,036.71	3,037.52	-2,929.69	-10,695.04	7,838.51
**Incremental expected life years**															
Mother lifetime	0.0175	0.0063	0.0355	-0.0125	-0.0326	0.0041	0.0050	-0.0038	0.0194	-0.0139	-0.0322	-0.0013	0.0035	-0.0021	0.0118
Offspring lifetime	0.0027	0.0000	0.0065	-0.0019	-0.0059	0.0007	0.0008	-0.0007	0.0035	-0.0022	-0.0059	0.0001	0.0005	-0.0004	0.0021
Combined lifetime	0.0202	0.0073	0.0409	-0.0144	-0.0373	0.0048	0.0058	-0.0044	0.0225	-0.0161	-0.0370	-0.0015	0.0041	-0.0024	0.0136
ICER per life year gained (mother only) (£)	3,398.02	-3,487.87	17,288.78	33,222.80	-92,539.48	107,066.92	3,482.56	-22,518.64	2,798.70	5,643.60	-5,007.68	53,474.74	-18,735.69	-104,364.26	106,519.86
ICER per life year gained (offspring only) (£)	49,056.38	-207,858.13	109,966.08	-196,503.31	-862,996.55	955,950.57	78,472.93	-611,518.59	209,450.28	24,094.32	-330,547.50	461,326.37	-162,246.28	-1,208,169.43	1,029,698.85
ICER per life year gained (combined) (£)	-11,027.55	-22,128.26	365.35	25,889.69	-93,525.63	79,175.40	-12,351.97	-39,591.11	-10,144.93	-9,361.30	-24,964.64	30,799.18	-30,613.64	-104,934.87	76,169.10
**Incremental expected QALYs**															
Mother lifetime	0.0483	0.0155	0.1041	-0.0345	-0.0939	0.0115	0.0138	-0.0103	0.0562	-0.0385	-0.0943	-0.0034	0.0098	-0.0057	0.0346
Offspring lifetime	0.0206	0.0036	0.0953	-0.0146	-0.0798	0.0033	0.0060	-0.0039	0.0405	-0.0164	-0.0817	-0.0008	0.0042	-0.0020	0.0256
Combined lifetime	0.0688	0.0211	0.1634	-0.0491	-0.1458	0.0162	0.0197	-0.0144	0.0841	-0.0549	-0.1468	-0.0048	0.0140	-0.0082	0.0511
ICER per QALY gained (mother only) (£)	1,278.91	-1,368.03	6,675.24	11,414.90	-34,895.82	40,707.36	-834.58	-9,595.15	984.69	1,406.68	-1,945.75	20,045.62	-7,131.56	-40,215.23	39,582.15
ICER per QALY gained (offspring only) (£)	-5,742.12	-24,910.39	13,210.56	300,433.12	-139,815.87	137,688.41	-11,382.74	-59,111.79	-2,005.38	-3,302.72	-29,957.16	63,567.89	-34,985.38	-174,174.78	128,821.45
ICER per QALY gained (combined) (£)	-3,501.42	-7,998.96	99.35	6,752.32	-30,873.87	24,671.85	-4,809.61	-14,322.99	-2,274.60	-3,376.39	-9,118.28	9,234.34	-9,982.56	-34,233.64	23,854.92
**Return on Investment**															
Expected intervention cost (£) *(Intervention - Comparator)*	173.60	170.03	177.08	-169.28	-172.87	-165.71	4.32	3.52	5.22	-143.72	-149.85	-137.09	29.88	24.75	35.54

Maternal healthcare cost (£) *(Comparator - Intervention)*	145.48	57.95	275.58	-103.90	-253.90	36.24	41.58	-30.73	159.23	-115.85	-251.19	-12.27	29.63	-17.51	95.47
Offspring healthcare cost (£) *(Comparator - Intervention)*	266.66	107.61	506.55	-190.55	-466.64	66.60	76.11	-56.46	292.57	-212.53	-463.28	-22.11	54.13	-32.49	172.21
Combined healthcare cost (£) *(Comparator - Intervention)*	412.14	169.99	765.25	-294.45	-709.52	103.59	117.69	-86.94	445.26	-328.39	-705.16	-34.12	83.76	-50.23	263.69
Cost-offset ratio	2.37	0.98	4.41	1.74	-0.61	4.19	27.54	-20.09	105.67	2.29	0.24	4.91	2.83	-1.69	8.94

Supplementary Information 10: Probabilistic analysis for the scenario analysis of no foetal loss: Scatterplot of incremental costs plotted against incremental QALYs for NHS SSS plus cessation-contingent financial incentive (CFIB) verses NHS SSS alone, NHS SSS plus Exercise (EB) or Text-message (TMB) based interventions versus NHS SSS plus CFIB, for combined mother and offspring over the lifetime and adulthood time horizon

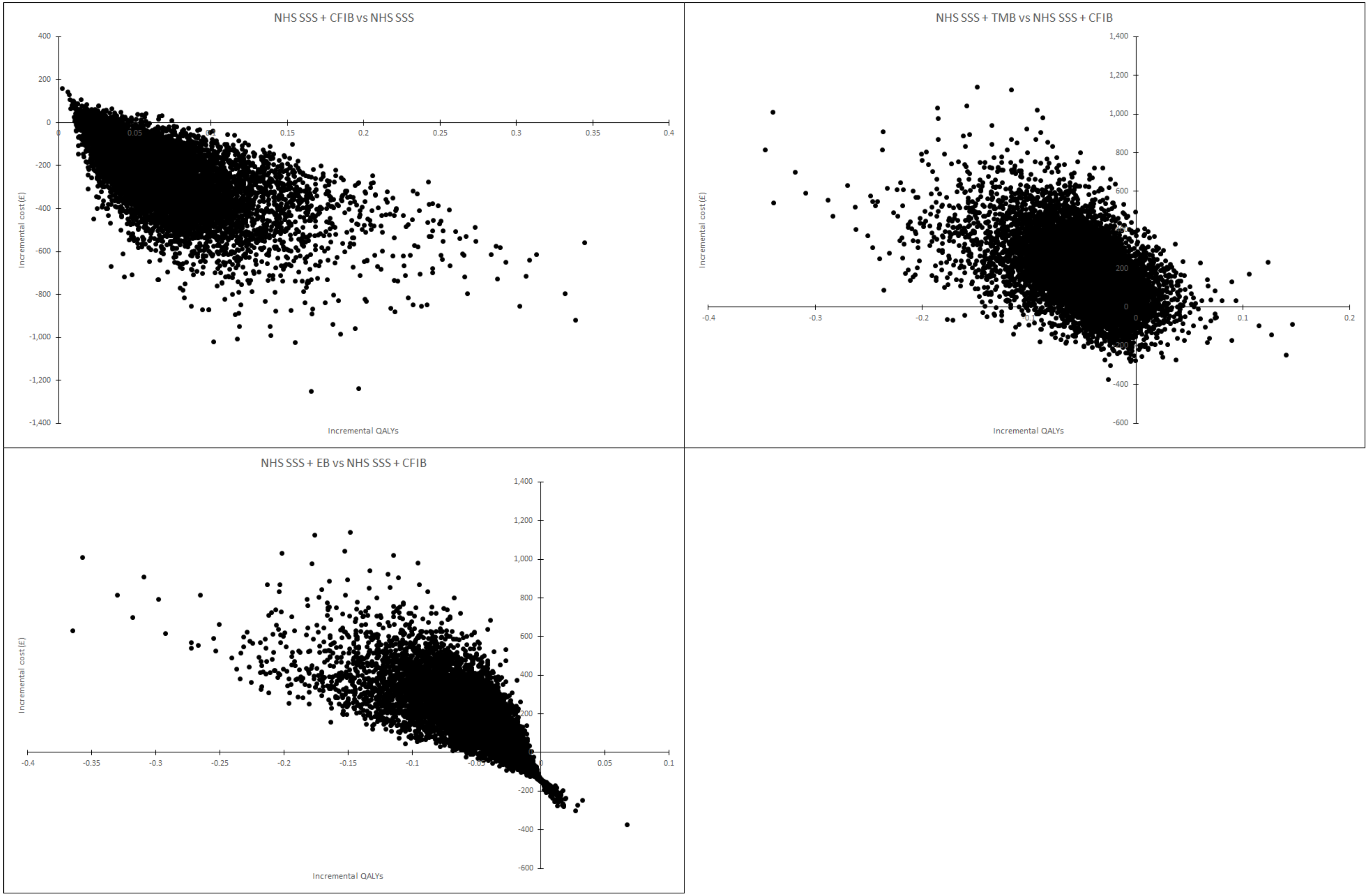

Supplementary Information 11: Probabilistic analysis for the scenario analysis of no foetal loss: Cost-effectiveness acceptability curve for cessation-contingent financial incentive (CFIB) versus NHS SSS alone (red line), NHS SSS plus Exercise (EB) (black line) or Text-message (TMB) (blue line) based interventions verses NHS SSS plus CFIB.

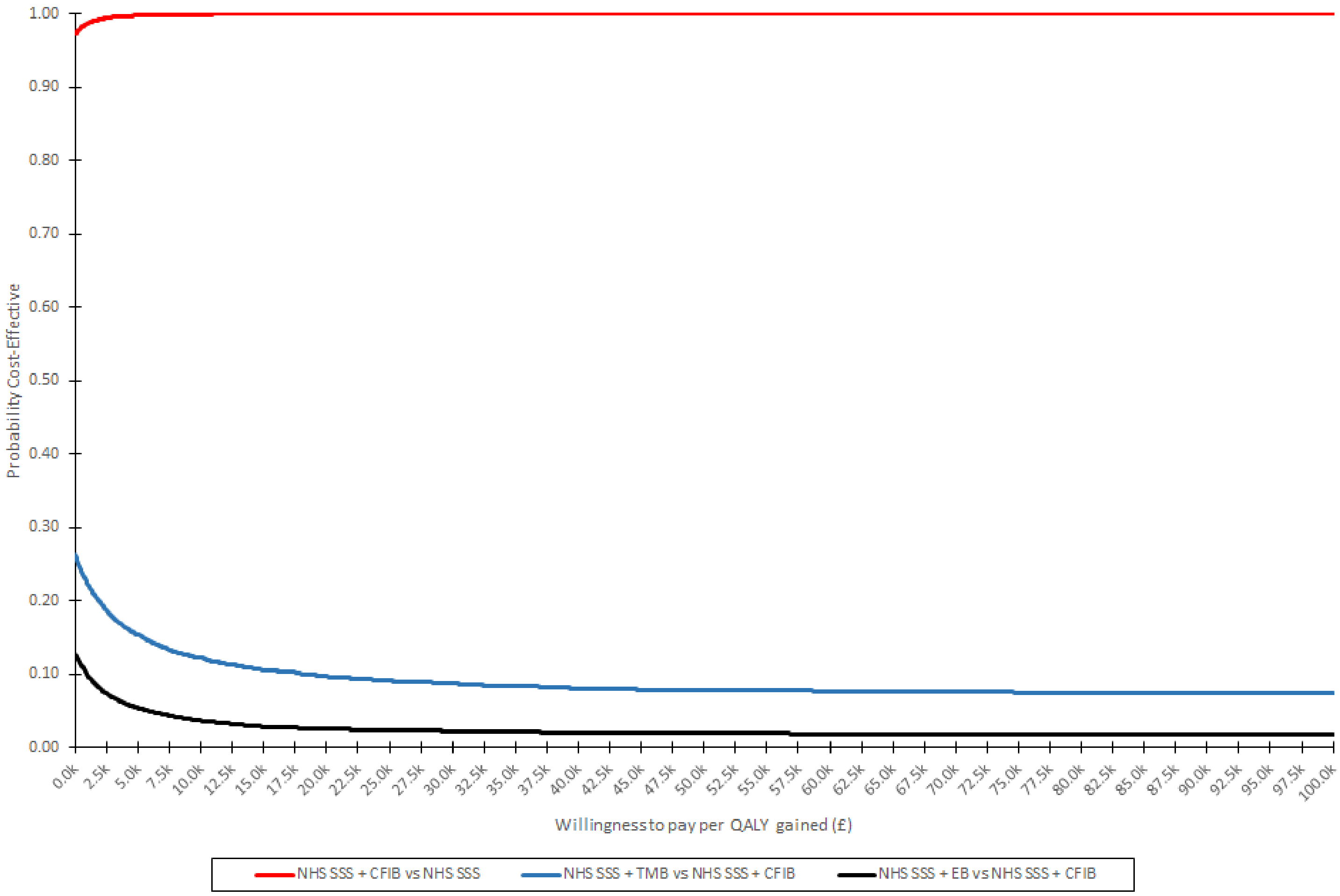

Supplementary Information 12: Scatterplot of NHS SSS + cessation-contingent financial incentives (CFIB) vs NHS SSS alone, for maternal outcomes over the lifetime using ESIP output from the primary analysis, for comparison with the original analysis of the CPIT trial

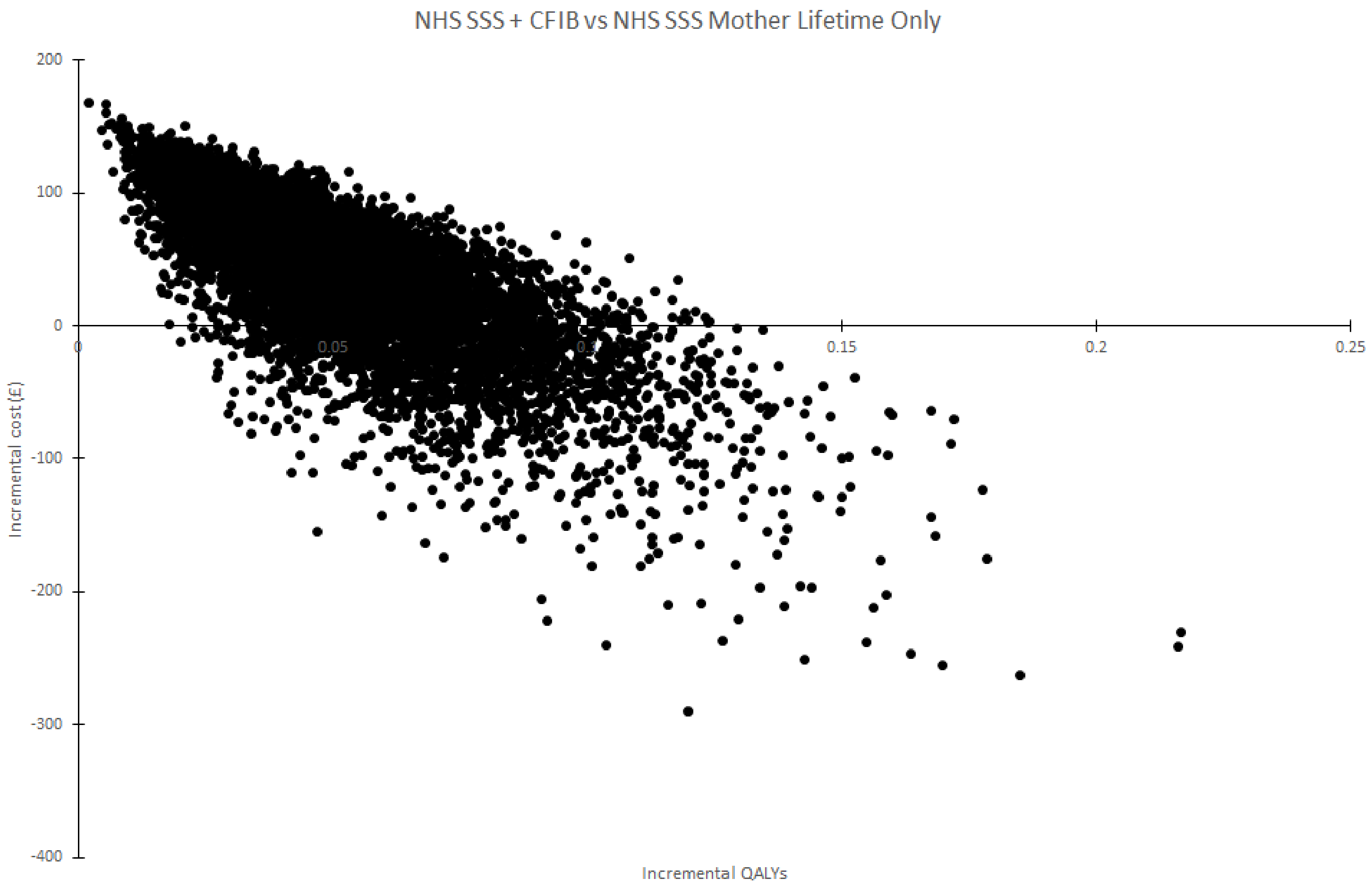

References

1. Jones M, Lewis S, Parrott S, Wormall S, Coleman T. Re-starting smoking in the postpartum period after receiving a smoking cessation intervention: a systematic review. Addiction (Abingdon, England). 2016;111(6):981-90.
2. Coleman T, Clark M, Welch C, Whitemore R, Leonardi-Bee J, Cooper S, et al. Effectiveness of offering tailored text message, self-help smoking cessation support to pregnant women who want information on stopping smoking: MiQuit3 randomised controlled trial (RCT) and meta-analysis 2021.
3. Ussher M, Lewis S, Aveyard P, Manyonda I, West R, Lewis B, et al. The London Exercise And Pregnant smokers (LEAP) trial: a randomised controlled trial of physical activity for smoking cessation in pregnancy with an economic evaluation. Health Technology Assessment (Winchester, England). 2015;19(84):vii-135.
4. Tappin D, Bauld L, Purves D, Boyd K, Sinclair L, MacAskill S, et al. Financial incentives for smoking cessation in pregnancy: randomised controlled trial. BMJ : British Medical Journal. 2015;350.
